# Perceived antidepressant efficacy associated with reduced negative and enhanced neutral mnemonic discrimination

**DOI:** 10.3389/fnhum.2023.1225836

**Published:** 2023-08-28

**Authors:** Taylor O. Phillips, Madelyn Castro, Rishi K. Vas, Lorena A. Ferguson, Amritha Harikumar, Stephanie L. Leal

**Affiliations:** Department of Psychological Sciences, Rice University, Houston, TX, United States

**Keywords:** antidepressants, emotion, memory, pattern separation, depression

## Abstract

**Introduction:**

While antidepressants are one of the first-line treatments for depression, the mechanisms underlying antidepressant action are unclear. Furthermore, the extent to which antidepressants impact emotional and cognitive dysfunction in depression requires more fine-grained approaches toward measuring these impacts in humans. Depression is associated with emotion and mood dysregulation in addition to cognitive deficits. Depressed individuals experience general memory impairment as well as a negativity bias in episodic memory, where negative events are better remembered than positive or neutral events. One potential mechanism hypothesized to underlie the negativity bias in memory is dysfunctional hippocampal pattern separation, in which depressed individuals tend to show impaired general pattern separation but enhanced negative pattern separation. Mnemonic discrimination tasks have been designed to tax hippocampal pattern separation in humans and provide a powerful approach to develop a mechanistic account for cognitive dysfunction in depression. While antidepressants have been examined primarily in rodent models in the context of hippocampal pattern separation, this has yet to be examined in humans.

**Methods:**

Here, we investigated how antidepressant usage and their perceived efficacy was associated with emotional mnemonic discrimination, given our prior work indicating a negativity bias for mnemonic discrimination in individuals with greater depressive symptoms.

**Results:**

We found that individuals who reported a greater improvement in their depressive symptoms after taking antidepressants (responders) showed reduced negative and enhanced neutral mnemonic discrimination compared to those with little to no improvement (non-responders). Perceived antidepressant efficacy was the strongest predictor of a reduction in the negativity bias for mnemonic discrimination, even when controlling for current depressive symptoms, antidepressant type, and other relevant factors.

**Discussion:**

These results suggest that antidepressants, when effective, can shift memory dynamics toward healthy function.

## 1. Introduction

Depression is associated with significant emotional, mood, and cognitive dysfunction (Nelson and Charney, 1981; Dere et al., 2010). Symptoms of depression include psychological symptoms, such as low mood, loss of interest, and poor concentration, and somatic symptoms, such as changes in appetite, lack of energy, sleep disturbance, and general aches and pains. Furthermore, depressed individuals also show general deficits in episodic memory, or memory for events and experiences, as well as a negativity bias in memory, where depressed individuals remember negative experiences better than neutral or positive experiences (Burt et al., 1995; James et al., 2021). Many types of medications and psychotherapy are currently available to treat depressive symptoms, which are generally shown to be more effective than controls (Barth et al., 2013; Cipriani et al., 2018). However, these treatments have significant limitations and challenges, thus, improving available treatments is critically important, especially given the large number of patients who fail to respond to treatment (Cuijpers, 2018).

Antidepressants are the first-line pharmacological treatment for depression. Selective serotonin reuptake inhibitors (SSRIs) are the most prescribed antidepressants, with monoamine oxidase inhibitor (MAOI), tricyclic (TCA) antidepressants, serotonin-norepinephrine reuptake inhibitors (SNRIs), norepinephrine-dopamine reuptake inhibitors (NDRIs), and other antidepressants used to a lesser extent (Dale et al., 2015; Luo et al., 2020; Bogowicz et al., 2021). Antidepressant development was based on the monoamine hypothesis of depression, suggesting that the underlying basis of depression is a depletion in serotonin, norepinephrine, and/or dopamine in the brain (Delgado, 2000). However, this classic hypothesis has been challenged and alternatives proposed (e.g., neuroplasticity hypothesis of depression) (Harmer et al., 2017; Liu et al., 2017; Moncrieff et al., 2022), highlighting the significant limitations in the overall understanding of the mechanism of action of antidepressants and their effectiveness in treating depressive symptoms, which are important factors to consider. Antidepressants have delayed onset of efficacy, intolerance issues, and limited response and remission rates, as antidepressants have been shown to be effective in roughly 50% of individuals (Taliaz et al., 2021). In fact, antidepressants appear to be most effective in those with greater depressive symptom severity (Kirsch et al., 2008; Fournier et al., 2010).

We know relatively little about how antidepressants work, why antidepressants have limited efficacy, and how antidepressants impact mood and cognitive function in depression, especially in human models. Notably, cognitive functions such as memory in individuals with depression are often overlooked, as antidepressants have been typically viewed as a treatment to alleviate primarily mood symptoms of depression. However, animal models have found substantial evidence that antidepressants can impact hippocampal function, in which the hippocampus is essential for episodic memory processing (Squire et al., 2004). Antidepressants can rescue hippocampal volume loss, stimulate the growth of new neurons in the dentate gyrus (DG) of the hippocampus, and reverse synaptic retraction between hippocampal subfields (Sousa et al., 2000; Sahay and Hen, 2007; Tartt et al., 2022). Given the influence that antidepressants can have on the hippocampus, it is important to evaluate the association between antidepressants and cognitive function, such as memory and emotional memory biases.

Mnemonic discrimination tasks have been developed to tax hippocampal pattern separation, a computation that processes similar experiences or events as unique using non-overlapping representations (Yassa and Stark, 2011). This framework has provided immense translational potential for clinical conditions with memory dysfunction, as the hippocampus is a major site of disruption across many brain disorders. Prior work has found that individuals with depression show impaired mnemonic discrimination for neutral stimuli (Shelton and Kirwan, 2013; Leal et al., 2014b) as well as enhanced mnemonic discrimination for negative stimuli when using an emotional mnemonic discrimination task (Leal et al., 2014b). The negativity bias in memory has traditionally been viewed as an overgeneralization of negative information (Fulford et al., 2012). However, mnemonic discrimination paradigms have yielded opposing results, such that discrimination of negative stimuli is enhanced in depression (e.g., greater detailed memory for negative experiences) and provides a novel mechanistic account for the negativity bias in memory in depression (e.g., underlying dysfunctional hippocampal pattern separation) (Leal and Yassa, 2018).

In the current study, we aimed to investigate the role of antidepressants on performance on an emotional mnemonic discrimination task in a sample of young adults (ages 18–35). Participants taking antidepressants (any type) for at least one month were eligible for the study. Given the limited efficacy of antidepressants, we measured perceived antidepressant efficacy to determine whether those who report a benefit from taking antidepressants (responders, R) may show larger impacts of antidepressants on memory performance than those who report little to no benefit from taking antidepressants (non-responders, NR). We hypothesized that greater perceived efficacy of antidepressants would be associated with less depressive symptoms and greater reductions in the negativity bias in memory (i.e., memory for negative relative to neutral events). We hypothesized that mnemonic discrimination measures would be more sensitive to these effects compared to target recognition. Based on prior findings in healthy and unmedicated depressed young adults (Leal et al., 2014b), we hypothesized that responders, relative to non-responders (controls), would show (1) enhanced neutral mnemonic discrimination and (2) reduced negative mnemonic discrimination.

## 2. Materials and methods

### 2.1. Participants

Participants ages 18–35 (*N* = 48) were recruited from Rice University and from the local Houston community through flyers, listserv, and website postings. The study lasted 2 h, and participants were compensated with either a $30 Amazon gift card ($15/h) or 2 Psychology course credits (1 credit/h) upon completion of the study. The study was carried out in accordance with relevant guidelines and regulations set forth by the Rice University Institutional Review Board (IRB). The full experimental protocol was approved by the Rice University IRB, and informed consent was obtained from all participants. Due to the COVID-19 pandemic, participants completed the study online via Zoom (October 2020–April 2021). All participants must have been actively taking antidepressants (regardless of the type of antidepressant and diagnosis) for at least one month prior to participation in the study. Most participants were diagnosed with depression and anxiety, which are highly comorbid (Kalin, 2020). Participants were split into responder (*N* = 21) and non-responder (*N* = 27) groups based on an arbitrary median split of a Perceived Antidepressant Efficacy score, discussed in detail below. Sample sizes are in line with previous research using the emotional mnemonic discrimination task in similar populations (Leal et al., 2014a,b). Participant demographics and questionnaire results can be found in Table 1.

**TABLE 1:** Participant demographics.

Group	Responder Mean (SD)	Non-responder Mean (SD)
*N*	21	27
Age	21.4 (2.8)	21.6 (3.7)
Sex	14F, 6M, 1T	20F, 5M, 2N
Race/ethnicity (%)		
White	66%	56%
Asian/Asian American	19%	22%
Black/African American	5%	7%
Multiracial	5%	11%
Other	5%	4%
Hispanic/Latinx/a/o	33%	19%
Education (completed)	13.6 years (2.2)	13.8 years (2.5)
Beck Depression Inventory-II (%)*	19.5 (11.2)	26.2 (11.6)
Healthy	33%	19%
Mild	24%	14%
Moderate	24%	26%
Severe	19%	41%
Depression diagnosis (%)	90%	89%
Depression + anxiety	52%	52%
Depression + anxiety + ADHD	14%	22%
Depression + ADHD	10%	0%
Depression	14%	15%
Anxiety	5%	4%
No diagnosis	5%	7%
Time taking antidepressants (%)		
1–4 months	10%	26%
4–6 months	14%	15%
6–12 months	14%	26%
12+ months	62%	33%
Switched antidepressants (past)	24%	33%
Number of depressive episodes (%)		
1–2 episodes	19%	22%
3–4 episodes	14%	19%
More than 5 episodes	7%	19%
More than 10 episodes	57%	37%
No response	0%	4%
Perceived depression severity (before antidepressants) *	8.9 (1.0)	6.5 (2.4)
Perceived depression severity (after antidepressants)	3.9 (1.2)	4.6 (1.8)
Perceived antidepressant efficacy (before – after)*	5.0 (1.0)	1.9 (1.4)
Antidepressant type (%)	12 SSRI, 9 other	16 SSRI, 11 other
SSRI	52%	60%
SNRI	5%	4%
NDRI	24%	25%
TCA	5%	11%
Other	5%	0%
Medications (%)		
Antidepressants	100%	100%
Antidepressants (secondary)	10%	11%
Stimulants	19%	11%
Antipsychotics	0%	4%
Anticonvulsants	5%	4%
Anxiolytics	5%	0%
Perceived Stress Scale (past month)	20.9 (4.3)	22.5 (5.1)
Beck Anxiety Inventory*	19.2 (10.1)	25.7 (10.2)
Hours of sleep (last night)	7.0 (1.5)	7.0 (1.8)
Hours of sleep (past month)	7.1 (1.1)	7.0 (1.5)
COVID-19 stress*	4.5 (0.9)	5.3 (0.7)
% stress level change due to COVID-19		
Gotten worse	76%	82%
Improved	10%	7%
Remained the same	14%	11%

*Significant group difference. F, female; M, male; T, transgener; N, non-binary; ADHD, attention-deficit/hyperactivity disorder; SSRI, serotonin-selective reuptake inhibitor; SNRI, serotonin and norepinephrine reuptake inhibitor; NDRI, norepinephrine and dopamine reuptake inhibitor; TCA, tricyclic antidepressant.

### 2.2. Questionnaires

Participants completed a demographics form to determine basic demographic information and a Medical Screening Questionnaire to determine medication use and exclude those with major neurological diseases. We developed an Antidepressant Questionnaire to measure various factors important to consider with antidepressant use, such as antidepressant type, duration taking antidepressants, number of depressive episodes prior to starting antidepressants, reported side effects, prior treatment history (e.g., switching antidepressant types), comorbid conditions (e.g., anxiety), as well as perceived antidepressant efficacy. Participants were asked to rate their depression on a scale of 1–10 (with 1 indicating “Not Depressed” and 10 indicating “Severely Depressed”) retrospectively (e.g., before they started antidepressant treatment) and their current state of depression (e.g., on antidepressant treatment). This approach was utilized since participants were already taking antidepressants prescribed by their doctors; thus, we did not manipulate antidepressant use in the current study to measure depressive symptoms or memory before and after starting antidepressants (e.g., this is an observational study). We determined perceived antidepressant efficacy for each participant by calculating a Perceived Antidepressant Efficacy score = (Depression score before taking antidepressants − current depression score while taking antidepressants). We examined this measure continuously as well as by group to stratify by responder status (responder and non-responder). We performed an arbitrary median split of the Perceived Antidepressant Efficacy score to create our responder and non-responder groups, in line with prior studies (Khan et al., 2004; Taliaz et al., 2021; Table 1). Participants with smaller reported change (0–3 point-change, scores ranging from −2 to 3) were grouped into the non-responder group and those with greater reported change (>3 point-change, scores ranging from 4 to 7) were grouped into the responder group.

Participants also completed the Beck Depression Inventory-II (BDI-II) (Beck et al., 1996) to measure current depressive symptoms, Beck Anxiety Inventory (BAI) (Beck et al., 1988) to measure current anxiety symptoms, the Pittsburgh Sleep Quality Index (PSQI) (Buysse et al., 1989) to measure various aspects of sleep, Perceived Stress Scale (PSS) (Cohen et al., 1983) to measure perceived stress in the past month, Subjective Memory Complaints Questionnaire (SMCQ) (Youn et al., 2009) to measure self-reported memory problems, Lifestyle and Exercise Questionnaire (LEQ) to measure various lifestyle factors such as cognitive, social, and physical activity, and diet, as well as a brief questionnaire about their experiences with COVID-19. At the time of participation, only four subjects had a past history of COVID-19. For the BDI-II, if a participant indicated suicidal thoughts (>1 on Question 9), participants were given the Suicidal Ideation Screening Questionnaire and were given resources to seek additional help. Scores from these questionnaires were included in regression models or as covariates, when relevant.

### 2.3. Emotional mnemonic discrimination task

**FIGURE 1:**
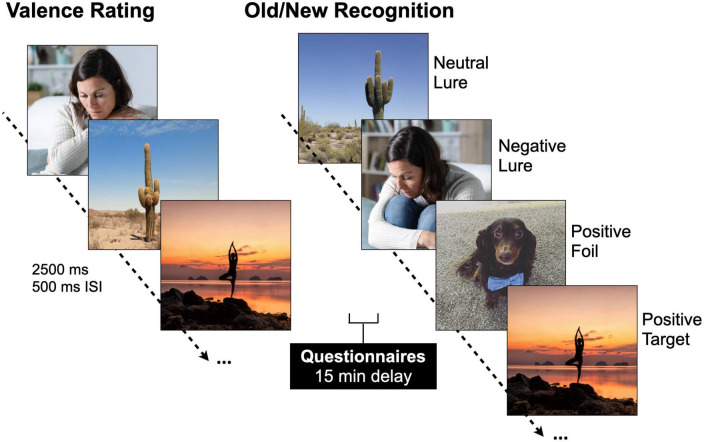
Emotional mnemonic discrimination task. Participants are shown negative, neutral, and positive stimuli [2,500 ms image, 500 ms inter-stimulus interval (ISI)] during encoding and are asked to rate the images as negative, neutral, or positive. After a brief 15-min delay, participants’ memory is tested with a recognition test where they are showing repeated images (targets), similar but not exactly the same images (lures), and new images (foils). Permission was obtained for the use of all images in this figure, in which the images are licensed by Shutterstock, available at https://www.shutterstock.com/, and one image which is an author’s personal photo (dog).

Participants completed an emotional mnemonic discrimination task (Figure 1) that includes negative, neutral, and positive scene stimuli (Leal et al., 2014b) and has been used broadly (e.g., Szőllősi and Racsmány, 2020; McMakin et al., 2022). The stimulus set has been well-validated, and each image has been previously rated for emotional valence, arousal, and similarity (Leal et al., 2014b). An Apple MacBook employing PsychoPy (version 3.2.4) was used to present the stimuli. During encoding and retrieval phases, images were presented for 2,500 ms. After each image, a fixation display was presented for 500 ms. Participants were shown 149 images during encoding that were either negative, neutral, or positive (7 min) and split evenly across emotion. Participants were instructed to rate the scene as negative, neutral, or positive via mouse click while the image was still on the screen. After encoding, participants completed the battery of questionnaires (discussed above) followed by a memory test. During retrieval, 291 images were shown including targets (repeated scenes), lures (similar scenes to those shown during encoding), and foils (new scenes). Lures were further divided into high and low similarity lures. All trial types were evenly distributed. Participants were asked to determine if a scene was exactly the same as one seen during encoding (“Old”) or if a scene was either new or different in some way (“New/Different”). It was made explicit that if an image was similar, but not identical to one seen before, they should choose “New/Different.” Participants received a short break halfway through the retrieval phase to minimize fatigue (each retrieval phase was 7 min).

Our two main memory measures of interest were target recognition and lure discrimination. Target recognition is a standard memory measure calculated using a discriminability index, *d′ = z(Hits) − z(False Alarms)*. Hits were the number of targets (old items) that were correctly recognized as “Old.” False alarms refer to the incorrect recognition of foils as “Old” and were subtracted to correct for response bias. Lure discrimination measures how well participants discriminate a similar lure image from one they had seen previously and taxes hippocampal pattern separation. Lure discrimination index (LDI) was calculated as *LDI = p(“New”| Lure) − p(“New”| Target)* and corrects for response bias (Yassa et al., 2011; Leal et al., 2014b). We calculated LDI for high and low similarity lures to examine whether level of similarity impacts mnemonic discrimination performance. We also calculated a negativity bias in memory score for each memory measure for each participant to examine individual differences in negative relative to neutral memory performance, calculated as *Negativity Bias (d′ or LDI)* = (Negative − Neutral) / (Negative + Neutral).

### 2.4. Statistical analyses

All statistical analyses were conducted in SPSS v. 28 (IBM Corp., Armonk, NY, USA). Planned comparisons were conducted using repeated-measures ANOVAs, *t*-tests (two-tailed), regression, and Pearson correlations. *Post-hoc* statistical tests for ANOVAs were corrected for multiple comparisons using Scheffe’s correction. All tests used the General Linear Model. Normality assumptions were investigated using Kolmogorov–Smirnov tests and all distributions investigated did not significantly deviate from the normal distribution. Repeated-measures tests were corrected for error non-sphericity using Greenhouse–Geisser correction. Effect sizes (*η_*p*_*^2^ and Cohen’s *d*) were reported when relevant. Statistical values were considered significant at a final corrected α level of 0.05, which appropriately controls for Type I error.

### 2.5. Data availability

The data generated in the current study are available in a GitHub repository.^1^

## 3. Results

### 3.1. Perceived efficacy of antidepressants associated with severity of initial depression and current depressive symptoms

**FIGURE 2:**
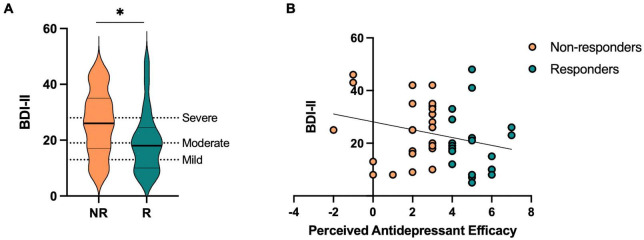
Current depressive symptoms in antidepressant responders (R) versus non-responders (NR) captured by perceived antidepressant efficacy. **(A)** Beck Depression Inventory-II (BDI-II) score across R and NR groups. **(B)** Correlation between perceived antidepressant efficacy and BDI-II in R and NR. Asterisk indicates significant group difference.

Participants were divided into responders and non-responders based on each participant’s Perceived Antidepressant Efficacy score [*t*(46) = 8.23, *p* < 0.001, Cohen’s *d* = −2.39]. On average, responders started out with more severe levels of depression prior to taking antidepressants compared to non-responders, which is consistent with studies suggesting antidepressants are more effective in those with more severe levels of depression (Table 1 and Supplementary Figures 1A, B; Kirsch et al., 2008; Fournier et al., 2010), and showed fewer current depressive symptoms as measured by the BDI-II compared to non-responders [*t*(46) = 2.02, *p* = 0.049, Cohen’s *d* = 0.59] (Table 1 and Figures 2A, B). We then conducted a stepwise linear regression to determine what factors significantly predict perceived antidepressant efficacy. We included gender, antidepressant type, duration of antidepressants, number of previous depressive episodes, history of switching antidepressants, perceived stress (past month), BDI-II, BAI, and hours sleep (past month and last night) as predictors of perceived antidepressant efficacy in the entire sample. This resulted in two significant models. In the first model, number of previous depressive episodes was the strongest predictor of perceived antidepressant efficacy (*r* = 0.44, β = 0.72, *p* = 0.002, *r*^2^ = 0.19), in line with previous work. In a second model, number of previous depressive episodes (β = 0.85, *p* < 0.001) and current depressive symptoms (BDI-II) (β = −0.06, *p* = 0.006; Figure 2B) significantly predicted perceived antidepressant efficacy (*r* = 0.57, *p* < 0.001, △*r*^2^ = 0.13).

### 3.2. Responders show reduced negative and enhanced neutral mnemonic discrimination

**FIGURE 3:**
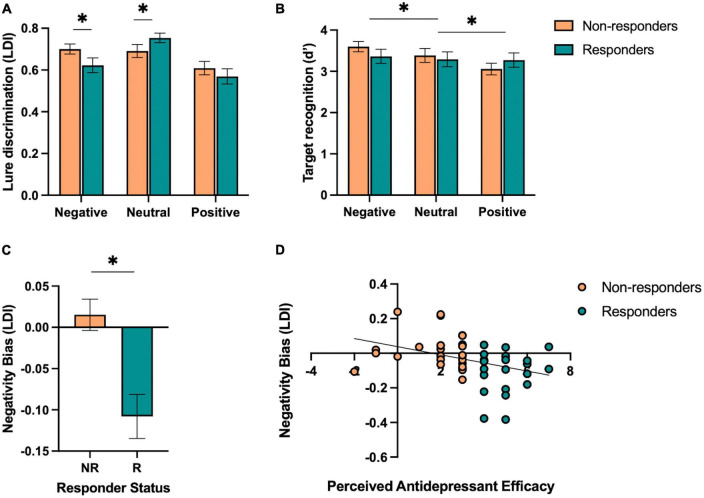
Performance on the emotional mnemonic discrimination task in antidepressant responders versus non-responders. **(A)** Low similarity lure discrimination index (LDI) performance across negative, neutral, and positive stimuli in responders (R) and non-responders (NR). **(B)** Target recognition performance across negative, neutral, and positive stimuli in R and NR groups. **(C)** Negativity bias in LDI (e.g., negative – neutral / negative + neutral) across R and NR groups. **(D)** Correlation between perceived antidepressant efficacy and negativity bias in LDI in R and NR. Asterisks indicate a significant interaction between emotion and responder status **(A)**, significant main effect of emotion **(B)**, and a significant group difference across responder status **(C)**.

Next, we aimed to test the hypothesis that responders, relative to non-responders, would show (1) enhanced neutral mnemonic discrimination and (2) reduced negative mnemonic discrimination given prior work in individuals with depressive symptoms showing deficits in neutral mnemonic discrimination and enhanced negative mnemonic discrimination (Leal et al., 2014b). First, we performed a repeated-measures ANOVA on lure discrimination with emotion (negative, neutral, and positive) and similarity (low and high) as within-subjects factors and responder status (responder and non-responder) as a between-subjects factor. We found a significant main effect of emotion [*F*(2,92) = 14.32, *p* < 0.001, *η_*p*_*^2^ = 0.24], where neutral lure discrimination was better than emotional lure discrimination [*F*(1,46) = 45.79, *p* < 0.001, *η_*p*_*^2^ = 0.50]. There was a significant effect of similarity [*F*(1,92) = 199.58, *p* < 0.001, *η_*p*_*^2^ = 0.81], where low similarity lures were better discriminated than high similarity lures. We also found a significant interaction between emotion and similarity [*F*(2,92) = 12.53, *p* < 0.001, *η_*p*_*^2^ = 0.21], where neutral lure discrimination was better than emotional lure discrimination for low similarity lures while negative was worse than neutral and positive lure discrimination for high similarity lures [*F*(1,46) = 20.54, *p* < 0.001, *η_*p*_*^2^ = 0.31]. Furthermore, we found a significant three-way interaction between emotion, similarity, and responder status [*F*(2,92) = 6.00, *p* = 0.004, *η_*p*_*^2^ = 0.12], where responders showed reduced negative and enhanced neutral lure discrimination compared to non-responders, but only for low similarity lures [*F*(1,46) = 9.93, *p* = 0.003, *η_*p*_*^2^ = 0.18; Figure 3A]. There was no main effect of responder status (*p*’s > 0.05).

For target recognition, we performed a repeated-measures ANOVA with emotion (negative, neutral, and positive) as a within-subjects factor and responder status (responder and non-responder) as a between-subjects factor. We found a significant main effect of emotion [*F*(2,92) = 3.42, *p* = 0.037, *η_*p*_*^2^ = 0.07], where a linear relationship was observed in which negative > neutral > positive target recognition [*F*(1,46) = 6.77, *p* = 0.012, *η_*p*_*^2^ = 0.13; Figure 3B]. There was no significant group difference or interaction between emotion and responder status (*p*’s > 0.05).

### 3.3. Perceived antidepressant efficacy predicts reduced negativity bias in lure discrimination

We also calculated a negativity bias score for each memory measure for each participant to consider individual differences in negative relative to neutral lure discrimination. At the group level, we found that responders showed a reduction in negativity bias for lure discrimination compared to non-responders [*t*(46) = 3.87, *p* < 0.001, Cohen’s *d* = 1.13; Figure 3C]. Next, we conducted a stepwise linear regression to determine what factors significantly predicted the negativity bias in lure discrimination. We included perceived antidepressant efficacy, gender, antidepressant type, duration of antidepressants, number of previous depressive episodes, history of switching antidepressants, perceived stress (past month), BDI-II, BAI, and hours of sleep (past month and last night) as predictors of the negativity bias in lure discrimination across the entire sample. Perceived antidepressant efficacy was the strongest predictor of the negativity bias in lure discrimination (*r* = 0.38, β = −0.02, *p* = 0.009, *r*^2^ = 0.14; Figure 3D). To determine whether current depressive symptoms moderated this effect, we conducted a moderation analysis with BDI-II as the moderator (*M*) between perceived antidepressant efficacy (*x*) and the negativity bias in lure discrimination (*y*). While the overall model was significant [*r*(3,44) = 0.41, *r*^2^ = 0.16, *p* = 0.046], there was no significant moderation of BDI-II on the relationship between perceived antidepressant efficacy and the negativity bias in depression [*F*(1,44) = 0.71, *p* = 0.41, △*r*^2^ = 0.013]. For target recognition, we found no difference between responders and non-responders for the negativity bias in target recognition [*t*(46) = 0.65, *p* = 0.52, d = 0.19], nor any significant predictors of the negativity bias for target recognition when conducting a stepwise linear regression with the same factors as noted above.

### 3.4. Exploratory analysis: antidepressant type may be differentially associated with emotional versus neutral lure discrimination

While we did not design the current study to include specific groups stratified by antidepressant type, we had a significant number of participants taking SSRIs (*N* = 27), as expected based on the frequency of prescriptions of SSRIs this type of antidepressant. While we had a relatively large number of participants taking SSRIs, we had fewer participants taking other types of antidepressants. Given that SSRIs impact primarily serotonin, while other antidepressants, such as SNRIs, NDRIs, and TCAs, target more than one neurotransmitter, we aimed to explore whether singular versus dual inhibitor mechanisms were associated with differential effects on emotional memory performance. In order achieve sufficient power to examine group differences, we collapsed the non-SSRI groups (SNRIs, NDRIs, TCAs, and other; *N* = 21) and performed *post hoc* analyses across those taking SSRIs and other antidepressants (α = 0.05, 1-β = 0.80, *d* = 0.83). However, interpretation of the other antidepressant group must be taken with caution given the heterogeneity of antidepressants in the sample, and future studies powered to examine the impact of different types of antidepressants more selectively will be essential.

**FIGURE 4:**
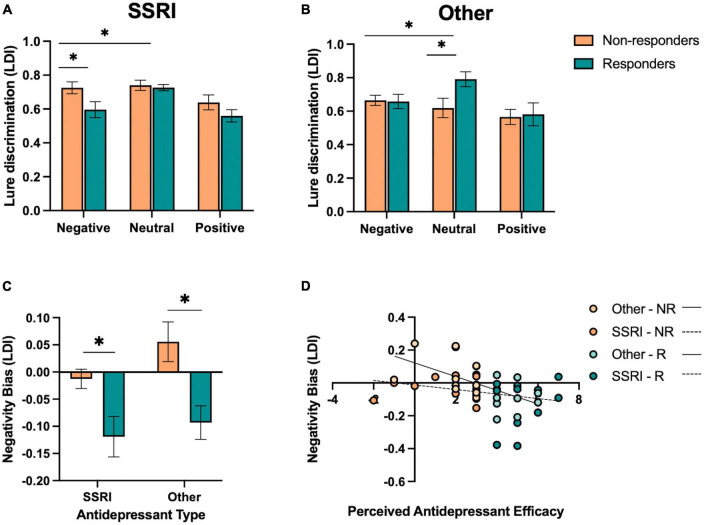
Effect of antidepressant type on emotional mnemonic discrimination performance in responders and non-responders. **(A)** Lure discrimination performance across negative, neutral, and positive stimuli for responders (R) and non-responders (NR) to selective serotonin reuptake inhibitors (SSRIs). **(B)** Lure discrimination performance across negative, neutral, and positive stimuli for R and NR groups to other types of antidepressants (SNRIs, NDRIs, and TCAs). **(C)** Negativity bias in LDI (e.g., negative – neutral / negative + neutral) across SSRI and other antidepressant groups, stratified by responder status. **(D)** Correlation between perceived antidepressant efficacy and negativity bias in LDI in SSRI and other antidepressant groups, stratified by responder status. Asterisks in panels **(A,B)** represent a significant interaction between emotion and responder status, and a significant effect of responder status in panel **(C)**.

Given our results in the full sample, we focused this exploratory analysis on lure discrimination, as this measure was most sensitive to emotion and responder status interactions. In those taking SSRIs, we performed a repeated-measures ANOVA for lure discrimination, with emotion (negative, neutral, and positive) as a within-subjects factor and responder status (responder and non-responder) as a between-subjects factor. We found a significant effect of emotion [*F*(2,52) = 9.78, *p* < 0.001, *η_*p*_*^2^ = 0.27], where those taking SSRIs showed better neutral versus emotional lure discrimination, but no significant effect of responder status or interaction between emotion and responder status (*p*’s > 0.05). Given the selectivity of effects for negative and neutral stimuli in our primary analysis, we focused our analysis on negative and neutral lure discrimination. We found a significant effect of emotion [*F*(1,26) = 10.42, *p* = 0.003, *η_*p*_*^2^ = 0.29], where those taking SSRIs showed better neutral versus negative lure discrimination overall. We also found a significant interaction between emotion and responder status [*F*(1,26) = 6.58, *p* = 0.016, *η_*p*_*^2^ = 0.20; Figure 4A], where SSRI responders showed reduced negative lure discrimination compared to non-responders.

We conducted the same set of analyses for those taking other antidepressants. We performed a repeated-measures ANOVA for lure discrimination, with emotion (negative, neutral, and positive) as a within-subjects factor and responder status (responder and non-responder) as a between-subjects factor. We found a significant effect of emotion [*F*(2,36) = 9.20, *p* < 0.001, *η_*p*_*^2^ = 0.34], where neutral was better than emotional lure discrimination [*F*(1,18) = 11.21, *p* = 0.004, *η_*p*_*^2^ = 0.38]. We also found a significant interaction between emotion and responder status [*F*(2,36) = 4.87, *p* = 0.015, *η_*p*_*^2^ = 0.21; Figure 4B], where, in contrast to those taking SSRIs, responders to other antidepressants showed enhanced neutral relative to emotional lure discrimination compared to non-responders [*F*(1,18) = 10.29, *p* = 0.005, *η_*p*_*^2^ = 0.36]. When focusing on negative versus neutral lure discrimination, we similarly find an interaction between emotion and responder status [*F*(1,18) = 10.22, *p* = 0.005, *η_*p*_*^2^ = 0.36], with enhanced neutral relative to negative lure discrimination in responders compared to non-responders.

When examining participants’ negativity memory bias score in an ANOVA across antidepressant type (SSRI and other) and responder status (responder and non-responder), we found a significant effect of responder status [*F*(1,44) = 15.91, *p* < 0.001, *η_*p*_*^2^ = 0.27; Figure 4C], where responders showed a reduction in the negativity bias in lure discrimination compared to non-responders. There was no main effect of antidepressant type or interaction between antidepressant type and responder status (*p*’s > .05). However, a *post-hoc t*-test revealed that there was a marginal effect of antidepressant type in the non-responders [*t*(25) = 1.85, *p* = 0.076, Cohen’s *d* = 0.09], where those taking other antidepressants showed a larger negativity bias in lure discrimination compared to those taking SSRIs.

Next, we examined the relationship between perceived antidepressant efficacy and the negativity bias in lure discrimination continuously within SSRI and other antidepressant groups. We found a significant correlation between perceived antidepressant efficacy and the negativity bias in lure discrimination in those taking other antidepressants (*r* = −0.58, *p* = 0.007; Figure 4D), and a weaker but non-significant relationship between these measures in those taking SSRIs (*r* = −0.25, *p* = 0.203; Figure 4D). We also conducted a moderation analysis with antidepressant type (*M*) as the moderator of the relationship between perceived antidepressant efficacy (*x*) and the negativity bias in lure discrimination (*y*). The overall model was significant [*r*(3,44) = 0.47, *r*^2^ = 0.23, *p* = 0.012], where perceived antidepressant efficacy significantly predicted the negativity bias in lure discrimination (β = −0.04, *p* = 0.006) and antidepressant type significantly predicted the negativity bias in lure discrimination (β = −0.13, *p* = 0.048), driven by the other antidepressant group, but no significant moderation of antidepressant type on the relationship between perceived antidepressant efficacy and the negativity bias in lure discrimination [*F*(1,44) = 2.51, *p* = 0.12, △*r*^2^ = 0.045].

## 4. Discussion

### 4.1. Perceived antidepressant efficacy impacts on emotional mnemonic discrimination

While antidepressants are one of the primary treatments for depression, the mechanisms underlying antidepressant action are unclear. Furthermore, the extent to which antidepressants impact emotional and cognitive dysfunction in depression requires more fine-grained approaches toward measuring these impacts in humans. Rodent models of chronic stress have found that antidepressants can improve hippocampal function and memory performance, thus, we aimed to determine whether antidepressants, when effective, could be associated with altered emotional mnemonic discrimination in humans, which relies on hippocampal pattern separation. We found that individuals who reported reduced depressive symptom severity after taking antidepressants (responders) showed reduced negative and enhanced neutral memory compared to those with little to no change in their depressive symptom severity (non-responders). Responders showed a reduction in the negativity bias in memory (e.g., negative relative to neutral memory performance). These effects were found at the group level (responders and non-responders) as well as when examining perceived antidepressant efficacy using continuous approaches (e.g., correlation and regression). Importantly, these results were selective to lure discrimination, which relies on hippocampal pattern separation as measured during high-resolution fMRI (Leal et al., 2014a), and not target recognition, suggesting that effective antidepressants may act on specific hippocampal computations (e.g., pattern separation) (Sahay et al., 2011; Gandy et al., 2017).

These results are in line with our hypotheses and prior work. While individuals with high levels of current depressive symptoms show a greater negativity bias in lure discrimination (Leal et al., 2014b), this negativity bias can be mitigated when taking antidepressants in those who perceive them to be effective in reducing their depressive symptom severity relative to non-responders. This was the case for reducing negative lure discrimination and enhancing neutral lure discrimination relative to non-responders, in which we hypothesized that this relationship would be evident based on prior work in individuals with depressive symptoms showing enhanced negative and reduced neutral lure discrimination compared to healthy young adult controls (Leal et al., 2014b). In fact, greater perceived antidepressant efficacy was associated with the largest reductions in the negativity bias in lure discrimination. This is in line with the literature suggesting that antidepressant treatment may provide the largest benefits to those with more severe depressive symptomology compared to more mild depressive symptoms (Kirsch et al., 2008; Fournier et al., 2010). It is important to note that our measure of perceived antidepressant efficacy is capturing how each participant feels antidepressants have alleviated their symptoms and may not be a reflection of their true depressive symptoms before starting antidepressants. However, we believe this only strengthens what this measure can tell us about memory in these individuals, such that greater change in depressive symptoms perceived by the participant was associated with a larger reduction of the negativity bias in lure discrimination, and no other measured factors significantly predicted this negativity bias. Thus, it will be important for future studies to determine whether examining change in BDI-II scores pre-antidepressant treatment and post-antidepressant similarly reflect this perceived antidepressant efficacy measure. Together, these results suggest that the dysfunctional memory effects we previously reported in unmedicated individuals with depressive symptoms could be alleviated when antidepressants are perceived to be effective.

Furthermore, we found that these effects were selective to low similarity lure discrimination. It could be that the high similarity lures were too difficult to discriminate, as all participants performed significantly worse on high compared to low similarity lures. We hypothesize that the emotional content of the images may not have been salient enough to modify memory performance for images with highly overlapping content (e.g., highest level of interference). This has been shown in other cognitively impaired individuals such as aging populations (Leal and Yassa, 2014); thus, antidepressants, either perceived to be effective or not, may not be able to alleviate memory deficits during high interference conditions. In healthy adults, the expected pattern of lure discrimination as a function of decreasing similarity is largely linear. However, in clinical conditions, one might expect to see variations in this pattern such that for certain similarity conditions, a clinical sample may show worse discrimination compared to controls at high or low similarity but may not differ from controls in the discrimination of targets or foils. Thus, differences in low versus high similarity may reflect an impairment that is selective to items with a certain level of interference and perhaps some specificity to pattern separation deficits.

### 4.2. Potential neurobiological mechanisms underlying the effect of perceived antidepressant efficacy on mnemonic discrimination

Prior work has suggested that the emotional mnemonic discrimination task is sensitive to hippocampal pattern separation, in which signals consistent with hippocampal pattern separation were found in the DG/CA3 subregions of the hippocampus during high-resolution functional neuroimaging (Leal et al., 2014a). These signals were found across both low and high similarity levels. Furthermore, a shift in amygdala-hippocampal dynamics was observed in individuals with depressive symptoms during emotional mnemonic discrimination, where hippocampal DG/CA3 subfield activity was reduced while amygdala activity was increased during negative lure discrimination relative to healthy controls (Leal et al., 2014a). The amygdala plays an important role in modulating memory, especially for emotional experiences. The basolateral amygdala (BLA) in particular may enhance processing of negative information that facilitates mnemonic discrimination in the absence of a normal DG/CA3 response (Leal et al., 2014a,^2017^). The shift in amygdala-hippocampal dynamics observed in depression could potentially be normalized in those taking antidepressants, when perceived to be effective, such that amygdala hyperactivity may be reduced and DG/CA3 activity may be increased during negative discrimination.

The DG/CA3 plays an important role in pattern separation, and impaired pattern separation is hypothesized to be a potential marker of reduced DG neurogenesis in individuals with depression (Tanti and Belzung, 2013; Gandy et al., 2017). Prior literature has found that antidepressant treatment can increase DG neurogenesis in animal models (Dranovsky and Hen, 2006), suggesting a potential mechanism of antidepressant action on hippocampal pattern separation in humans. Furthermore, increasing DG neurogenesis has been shown to improve hippocampal pattern separation as well as reduce depressive-like behaviors in rodent models (Sahay et al., 2011; Hill et al., 2015). This would need to be explored in humans using high-resolution imaging methods to determine the underlying neurobiological mechanisms supporting the shift in negative and neutral mnemonic discrimination with perceived antidepressant efficacy. Based on the findings discussed above, DG activity and neurogenesis is reduced in depression, however, behaviorally, individuals with depression show enhanced negative and impaired neutral mnemonic discrimination, in which the BLA may more heavily impact negative memory (e.g., amygdala hyperactivity in depression associated with enhanced negative mnemonic discrimination) (Leal et al., 2014a). Thus, antidepressant effects on the brain may be multifaceted, such that they increase DG neurogenesis and activity leading to rescued general memory but may also reduce BLA hyperactivity (Young et al., 2020) leading to reduced negative mnemonic discrimination.

Regarding antidepressants, there has been work showing that adult hippocampal neurogenesis is required for some, but not all, of the behavioral effects of antidepressants (David et al., 2009). It has been shown that increasing adult hippocampal neurogenesis is sufficient to impact pattern separation (Hill et al., 2015) and depression-like behaviors (Sahay et al., 2011). However, the presence of adult human neurogenesis has been significantly debated (Kumar et al., 2019), and it is unclear what role neurogenesis may play in hippocampal pattern separation (Johnston et al., 2016). There has been mixed support for neurogenesis as a perceptible mechanism in the adult human brain and a lack of consensus on the location of adult human neurogenesis in the brain due to anatomical differences across species (Kumar et al., 2019). While there is evidence of adult hippocampal neurogenesis in humans (Kempermann et al., 2018), it is primarily examined in post-mortem tissue. However, novel neuroimaging techniques such as magnetic resonance spectroscopy (MRS) may be useful in solving this limitation, which has the potential to measure signals consistent with hippocampal neurogenesis in humans (e.g., a metabolic biomarker for the detection and quantification of neural stem and progenitor cells in the human brain *in vivo*) (Manganas et al., 2007). While these methods are currently under development, they may provide important insight into the neural mechanisms underlying antidepressant action on the hippocampus in humans.

Furthermore, there is evidence to suggest that hippocampal DG and CA3 subfields are especially impacted in depression (Malykhin and Coupland, 2015; Samuels et al., 2015), and antidepressants may selectively target particular hippocampal subfields more than others (Huang et al., 2013; Tai et al., 2021). However, to our knowledge, no studies have examined the impact of antidepressants on hippocampal pattern separation in humans. Thus, this study provides the first step in establishing this paradigm as a sensitive task in detecting subtle changes in memory that depends on the emotional context of the experience and provides important preliminary data to suggest that (1) perceived antidepressant efficacy is important to consider when examining antidepressant effects on memory, and (2) antidepressants that target different neurotransmitters systems may have differential impacts on memory and MTL function.

It is important to note that alterations in the hippocampus under chronic stress are reversible. Chronic stress can lead to a retraction of hippocampal CA3 dendrites (Conrad, 2006) and a decrease in DG neurogenesis in rodents (McEwen and Magarinos, 2001). Antidepressants can reverse these synaptic changes by increasing DG neurogenesis and by increasing CA3 dendritic arborization (Baj et al., 2012; Castrén and Hen, 2013; Hill et al., 2015). Notably, these hippocampal subregions are essential in performing pattern separation. Thus, our results suggest that antidepressants may be effective at altering these hippocampal mechanisms in humans but may depend on a number of factors such as perceived efficacy, number of previous depressive episodes, and antidepressant type.

### 4.3. Potential impact of different types of neurotransmitters on emotional memory and MTL function

We also found that antidepressant type may play an important role in altering emotional versus neutral memory. SSRIs appeared to selectively target emotional memory (e.g., reduction in the negativity memory bias) while other types of antidepressants selectively targeted neutral memory (e.g., enhancement in neutral lure discrimination). While these analyses were exploratory, there may be important differences in how antidepressant type may impact performance on emotional versus neutral memory given their different monoamine targets. Rodent studies have found that SNRIs, but not SSRIs enhance recognition memory (Feltmann et al., 2015). Evidence from human studies suggests that SNRIs may be more beneficial for cognitive function compared to other antidepressants (Harmer et al., 2003; Biringer et al., 2009). This could explain why we see an enhancement in neutral lure discrimination in those taking other kinds of antidepressants including SNRIs. While SSRIs may not enhance general memory, our results suggest that they may reduce memory for negative experiences. Thus, the content and emotional significance of the memory is important to consider when examining whether antidepressant type influences cognition in different ways.

## 5. Limitations

It is important to note that there are limitations to the current study. First, this study was observational in nature; thus, we did not measure memory and depressive symptoms before starting antidepressants and at various timepoints after taking antidepressants. The goal of the current study was to determine if there might be measurable effects of antidepressants on memory when considering perceived antidepressant efficacy. Our results support this hypothesis, paving the way for future studies to explicitly manipulate, measure, and track the impact of antidepressants on memory and depressive symptom severity. It will be important to establish baseline memory and depressive symptoms and track progress of antidepressant effectiveness at various timepoints (e.g., after 1 month, 6 months, and 1 year).

It will also be important to determine whether perceived antidepressant efficacy scores are associated with objective and clinical measures of depressive symptoms pre- and post-antidepressant treatment, and perhaps examine which types of symptoms were most improved. Based on clinical standards (>50% reduction in depressive symptoms), 81% of the responder group reached clinical efficacy, which is consistent with other studies quantifying responder status (Taliaz et al., 2021). While our perceived antidepressant efficacy measure was not designed with clinical thresholds in mind, it will be important for studies examining treatment efficacy to consider how responder status is characterized, which can vary widely across studies and measures of symptom severity (Taliaz et al., 2021). Here, we applied an arbitrary median split to determine responder status to maximize group differences and have large enough samples per group; however, there are many approaches and measures that may provide a more accurate characterization of responder status. While examining perceived efficacy of antidepressants continuously alleviates this problem to some degree, the threshold for responsiveness to antidepressant treatment remains a critical issue to address in studies examining change in symptomology and severity due to treatment.

It is important to note that it is not expected that all responders to antidepressants (as defined in this study, or even in general) would be considered “healthy” again. On average, the responder group showed significant reductions in depressive symptoms compared to non-responders, but the range of depression scores varied both groups. In this study, we found no significant contribution of depressive symptoms interacting with perceived antidepressant efficacy on the negativity bias in lure discrimination, however, this remains an important factor to consider in future studies to better understand how treatment interacts with current depressive symptomology. In our sample, 40% of the responder group had a BDI <15 while only 20% of the non-responder group had a BDI <15. Thus, while the responder group had twice as many participants who would be considered below previously used thresholds of clinically relevant depressive symptoms (Sprinkle et al., 2002; Leal et al., 2014b), there was still a large amount of variability in level of current depressive symptoms (Figure 2). Here, our responder groups are reflecting perceived efficacy of antidepressants, not absolute level of depressive symptoms, which is an important concept to consider when evaluating efficacy of antidepressants.

Second, the current study focused on the examination of those taking antidepressants, using non-responders as controls, and indirectly compared relative performance to prior findings in a healthy control and unmedicated depressed sample (Leal et al., 2014b). While the current findings were in line with this prior work, in which non-responders’ performance showed a similar pattern across emotional memory to unmedicated depressed participants while responders’ performance showed a similar pattern across emotional memory to the healthy control sample, future work aiming to replicate these prior findings and directly compare these samples to those taking antidepressants will be important.

Third, we did not design the study to investigate the impact of different types of antidepressants, which could have differential impacts on memory and MTL given their varying neurotransmitters of target. From our exploratory analysis of antidepressant type, there appears to be differential effects of different antidepressant types on emotional memory that will be important for future studies to consider. We also included individuals taking antidepressants for any diagnosis or co-morbid diagnoses, given that depression is often comorbid with anxiety and other neuropsychological disorders (Kalin, 2020). Most of our sample was diagnosed with depression and co-morbid anxiety, as expected, but it will be important for future studies to determine whether antidepressants may be effective for those with only one diagnosis and how comorbid conditions may further interact with antidepressant efficacy. It is important to note that we included BAI in our regression models but found no significant impacts of current anxiety symptoms on our measures of interest. Moreover, roughly 30% of the non-responder group and 42% of the responder group were also taking other medications (e.g., stimulants, anticonvulsants, antipsychotics, and anxiolytics) concurrently with an antidepressant (see Table 1). Additional medication usage may explain some of the variance in antidepressant efficacy and memory performance, however, it was a very small percentage of participants taking various combinations of multiple medications. Future studies would benefit from recruiting participants taking only antidepressants to determine whether it is specifically antidepressants that are leading to changes in efficacy and memory performance or whether combinatory treatments (“polypharmacy”) may lead to better outcomes (Millan, 2014). However, given the heterogeneity of psychiatric diagnosis and medication use, limiting samples to singular medication use may not accurately reflect the general population.

Fourth, this study was conducted during the height of COVID-19 pandemic, which could have impacted levels of stress and current depressive symptoms in our sample (see Table 1). The study was conducted over Zoom, which could introduce greater variability in the experimental setting. However, we ensured participants were situated in a quiet room with a computer with a camera and that internet connectivity was stable to minimize any confounding factors. Finally, neurobiological data must be acquired to determine whether antidepressants impact hippocampal pattern separation in humans. An important future direction will be utilizing high-resolution imaging techniques capable of probing these hippocampal subfields and computations. Our emotional mnemonic discrimination task provides a more sensitive measure of memory compared to standard memory tests to gain insight and a higher level of precision into how antidepressants may be impacting hippocampal subfields and amygdala subnuclei important for memory processing in humans (Stark et al., 2013; Leal and Yassa, 2018).

While antidepressants are the primary pharmacological treatment for depression (Cipriani et al., 2018), we know relatively little about how antidepressants work and how they impact cognition and mood (Harmer et al., 2017; Liu et al., 2017; Moncrieff et al., 2022). Rodent studies have begun to unpack the complexity of antidepressants and how they might influence memory (Lino de Oliveira et al., 2020) but human studies are only beginning to understand how antidepressants modulate both cognitive and mood symptoms of depression. The utility of pattern separation as a construct can further our understanding of basic memory processing as well as neuropsychiatric diseases such as depression (Leal and Yassa, 2018). Thus, this study provides novel insight into the potential underlying neural mechanisms of antidepressant action on memory and MTL function and highlights the importance of taking responsiveness to antidepressant treatment into account when examining how antidepressants impact mood and cognition.

## Data availability statement

The datasets presented in this study can be found in online repositories. The names of the repository/repositories and accession number(s) can be found in the article.

## Ethics statement

The studies involving humans were approved by the Rice University Institutional Review Board. The studies were conducted in accordance with the local legislation and institutional requirements. The participants provided their written informed consent to participate in this study. Written informed consent was obtained from the individual(s) for the publication of any potentially identifiable images or data included in this article.

## Author contributions

TP involved in investigation, formal analysis, data curation, and writing—original draft. MC involved in data curation and writing—review and editing. RV involved in investigation and writing—review and editing. LF and AH involved in project administration and writing—review and editing. SL involved in conceptualization, formal analysis, writing—review and editing, visualization, supervision, and funding acquisition. All authors reviewed and revised the final manuscript.

## Footnotes

1
https://github.com/lealmemorylab/antidepressants
