# Supplementary material for: Perceived antidepressant efficacy associated with reduced negative and enhanced neutral mnemonic discrimination
Source: Front Hum Neurosci. 2023 Aug 28;17:1225836. doi: 10.3389/fnhum.2023.1225836 (PMC10494429; doi:10.3389/fnhum.2023.1225836)
Supplement: Supplementary file 1 [file Image_1.pdf]

Supplementary Material

Perceived antidepressant efficacy associated with reduced negative and enhanced neutral mnemonic discrimination

Taylor O. Phillips**, Madelyn Castro**, Rishi K. Vas, Lorena A. Ferguson, Amritha Harikumar, & Stephanie L. Leal*

***Correspondence:** Stephanie L. Leal, Ph.D.: stephanieleal@rice.edu

1 Supplementary Figures and Tables

1.1 Supplementary Figures

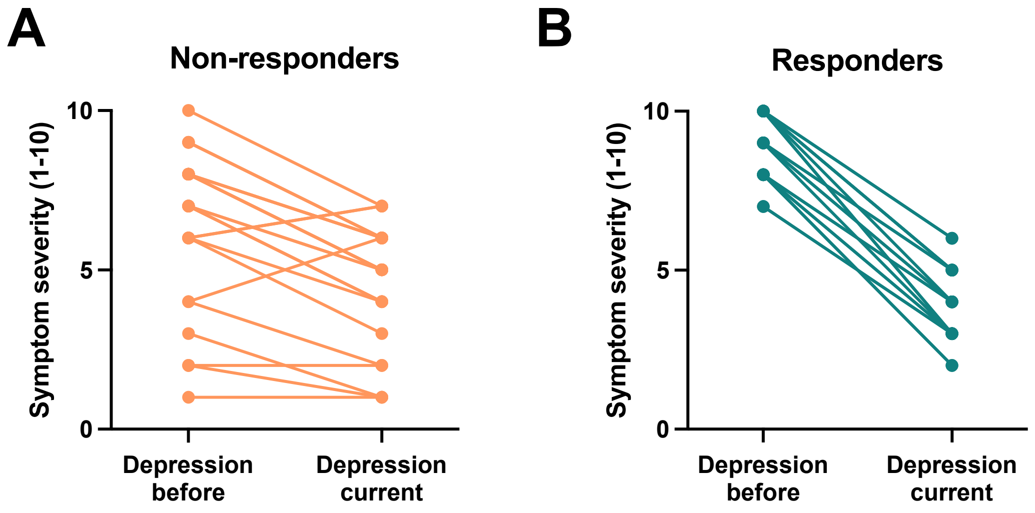

Supplementary Figure 1. Responders versus non-responder differences in depressive symptom severity. A) Non-responders depressive symptom severity before (retrospectively) versus current, B) Responders depressive symptom severity before (retrospectively) versus current.
